# Safety Assessment and Biological Effects of a New Cold Processed SilEmulsion for Dermatological Purpose

**DOI:** 10.1155/2013/181634

**Published:** 2013-11-05

**Authors:** Sara Raposo, Ana Salgado, Lídia Gonçalves, Pedro C. Pinto, Manuela Urbano, Helena M. Ribeiro

**Affiliations:** ^1^Research Institute for Medicines and Pharmaceutical Sciences (iMed.UL), Faculty of Pharmacy, University of Lisbon, Avenida Prof. Gama Pinto, 1649-003 Lisbon, Portugal; ^2^Laboratório Edol Produtos Farmacêuticos S.A., Estrada Casal Canas Lote 6, 2790-007 Carnaxide, Portugal

## Abstract

It is of crucial importance to evaluate the safety profile of the ingredients used in dermatological emulsions. A suitable equilibrium between safety and efficacy is a pivotal concern before the marketing of a dermatological product. The aim was to assess the safety and biological effects of a new cold processed silicone-based emulsion (SilEmulsion). The hazard, exposure, and dose-response assessment were used to characterize the risk for each ingredient. EpiSkin assay and human repeat insult patch tests were performed to compare the theoretical safety assessment to *in vitro* and *in vivo* data. The efficacy of the SilEmulsion was studied using biophysical measurements in human volunteers during 21 days. According to the safety assessment of the ingredients, 1,5-pentanediol was an ingredient of special concern since its margin of safety was below the threshold of 100 (36.53). EpiSkin assay showed that the tissue viability after the application of the SilEmulsion was 92 ± 6% and, thus considered nonirritant to the skin. The human studies confirmed that the SilEmulsion was not a skin irritant and did not induce any sensitization on the volunteers, being safe for human use. Moreover, biological effects demonstrated that the SilEmulsion increased both the skin hydration and skin surface lipids.

## 1. Introduction

Over the years, research has focused on strategies to optimize the potency of topical steroids while minimizing adverse effects. Several attempts have been made to increase the safety of topical glucocorticoids (TG) treatment, including new application schedules, special vehicles, and newly synthesized agents. The key factors in the management of anti-inflammatory topical diseases are not only related to the use of effective topical anti-inflammatory agents but also in providing skin hydration and barrier repair [1].

Emulsion systems used in dermopharmacy have to fulfill a number of requirements, for example, acceptable physical stability, chemical inertness, satisfactory safety profile, and an attractive appearance, and also provide an appropriate carrier system for the active agent. The system must be nonirritant to the skin, easily applied, and removed from the skin [2]. In order to provide all of these attributes to emulsions, several excipients have to be used [3] such as surfactants, coemulsifiers, polymers, preservatives, emollients, and solubilizers. It is of crucial importance to evaluate the safety profile of the ingredients used in such vehicles especially if those vehicles are to be applied on damaged skin.

Dermatological emulsions without drug are considered cosmetic products falling under the general requirements of the EC Cosmetics Directive 76/768 [4] regarding their safety. This directive will be replaced stepwise by the new EC Cosmetics Regulation 1223/2009 [5]. Under both regulations, the toxicological profile of all used ingredients and detailed knowledge of the product-specific exposure are required as they are fundamental for the safety assessment [6]. As imposed by the legislation, cosmetics are considered to be safe for the consumer. Although this appears to be self-evident, there is a whole scientific exercise preceding this “obvious” conclusion. The safety of a cosmetic product is determined based on the safety assessment of its ingredients, which is done using the literature data, *in vitro* tests, and human tests since, in EU, finished cosmetic products are no longer tested in animals [7].

There are ingredients of special concern in terms of safety assessment like preservatives, solubilizers, and surfactants. Concerning the surfactants, most of them are based on ethoxylated nonionic emulsifiers or their mixtures with long chain fatty alcohols (so called mixed emulsifiers). While vehicles based on these mixed emulsifiers meet general requirements for pharmaceutical bases, their use may be accompanied by adverse skin reactions [8].

Due to the excellent barrier nature of the *stratum corneum* (SC), human external contact with a substance rarely results in its penetration through the skin with a significant systemic exposure; however, skin care products produce local exposure. Therefore, human systemic exposure to ingredients can rarely be completely excluded [9].

In this work, we aimed to evaluate the safety profile and the biological effects of a new cold processed SilEmulsion intended to be used as a vehicle for corticoids or as an adjuvant in topical anti-inflammatory therapy. The safety evaluation was performed using literature data and a systematic approach for the safety assessment, comparing it with *in vitro *and *in vivo *data obtained by the methods of skin bioengineering and human volunteers, respectively.

## 2. Materials and Methods

### 2.1. Materials

1,5-Pentanediol, 99%, and hydroxypropyl methylcellulose (HPMC) were obtained from Sigma Aldrich, Germany; bis-PEG/PPG-16/16 PEG/PPG-16/16 dimethicone (and) caprylic/capric triglyceride (Abil Care 85); PEG-20 glyceryl laurate (Tagat L2), C12–15 alkyl benzoate (Tegosoft TN); and cetrimide BP and isopropyl myristate (Tegosoft M) were a gift from DS Produtos Químicos, Portugal; methyl vinyl ether/maleic anhydride copolymer cross-linked with decadiene (PVM/MA), (Stabileze QM) was purchased from ISP, Germany.

### 2.2. Preparation of o/w Cold Processed Emulsion

The SilEmulsion was created as described elsewhere [3]; briefly, it was prepared at room temperature as an oil liquid phase, achieved by dissolving the Bis-PEG/PPG-16/16 PEG/PPG-16/16 dimethicone (and) caprylic/capric triglyceride and the coemulsifier (PEG-20 glyceryl laurate) into the oils (C12–15 alkyl benzoate and isopropyl myristate) and mixing (Helipath 130 rpm) for about 30 minutes.

Next, an aqueous phase was prepared at room temperature by dispersing the aqueous thickening agents (HPMC and PVM/MA) in water. Cetrimide and the 1,5-pentanediol were added to the aqueous solution, and the resulting mixture was homogenized until a clear homogeneous gel was achieved.

The emulsification phase was performed at room temperature by slowly adding the oil phase to the aqueous phase with high shear mixing at a rate about 12800 rpm/min (IKA T25 ULTRA TURRAX). This addition was done at uniform rate over a period of 5 minutes.  Table 1 describes the main function of the ingredients present in the SilEmulsion as well as the qualitative and quantitative composition.

### 2.3. Physical and Microbiological Stability of the SilEmulsion

The experimental protocol was based on the guideline stability testing of existing active substances and related finished products [23]. One batch of the SilEmulsion was produced and was then stored for 12 months at room temperature (real time, 25 ± 2°C/60% ± 5% humidity) and at accelerated aging conditions (oven at 30 ± 2°C/65% ± 5% relative humidity and 40 ± 2°C/75% ± 5% relative humidity). Samples were taken for analysis at the end of the following time periods: 0, 1, 3, 6, and 12 months and assessed in terms of macroscopic organoleptic characteristics, pH value (Metrohm pH Meter 744), and apparent viscosity (Brookfield RV DV-II, SSA, spindles SC4-21 and SC4-27).

The microbiological stability assessment was performed according to the Portuguese Pharmacopoeia [24].

### 2.4. Safety Assessment of the SilEmulsion

The safety evaluation of the SilEmulsion was conducted according to the SCCS's Notes of Guidance for Testing of Cosmetic Ingredients and their Safety Evaluation [25].

For each ingredient, data was acquired from ingredient's supplier and publicly available literature.

#### 2.4.1. Hazard Identification

Based on the results of *in vivo* tests, *in vitro* tests, clinical studies, and human epidemiological studies, the intrinsic physical, chemical, and toxicological properties of each ingredient under consideration were studied to identify whether the substance has the potential to damage human health.

#### 2.4.2. Exposure Assessment

The amount and the frequency of human exposure to the SilEmulsion were determined. The systemic exposure dose (SED) was calculated for each ingredient, according to (1). Consider

(1) $$\begin{array}{c} \text{SED}\;=\;A\left(\text{mg}/\text{kg}\;\text{bw}/\text{day}\right)\times \frac{C\left(\%\right)}{100}\times \frac{\text{DA}\left(\%\right)}{100}, \end{array}$$

where *A* is the estimated daily exposure to a cosmetic product per Kg body weight (bw), based upon the amount applied and the frequency of application; *C* is the concentration of the ingredient under study in the finished cosmetic product; and DA is the dermal absorption expressed as a percentage of the test dose assumed to be applied in real life conditions.

#### 2.4.3. Dose-Response Assessment

The relationship between the toxic response and the exposure was studied. Public data was used to find out the no observed adverse effect level (NOAEL) which is the highest dose or exposure level where no adverse treatment-related findings are observed.

#### 2.4.4. Risk Characterization

The probability that the substances under investigation cause damage to human health and the level of risk were examined. In the case of a threshold effect, the margin of safety (MoS) was calculated according to (2). Consider

(2) $$\begin{array}{c} \text{MoS}=\frac{\text{NOAEL}}{\text{SED}}. \end{array}$$

### 2.5. EpiSkin Assay

The validated reconstructed human epidermis EpiSkin skin irritation test method was used [26].

The EpiSkin tissues were supplied by SkinEthic Laboratories (http://www.skinethic.com/) consisting of a reconstructed organotypic culture of adult human keratinocytes developed into a multilayered and well-differentiated epidermis.

The experiment was performed following manufacturer's protocol. The 12-well plates containing 12 inserts of tissues (0.38 cm^2^) were transferred into 12 wells plates containing 2 mL of maintenance medium and incubated at 37°C (5% CO_2_, >95% humidity). After 24 h, the second column of each plate was filled with maintenance medium preheated at 37°C.

Ten mg of the SilEmulsion was applied directly for a duration of 15 minutes to the epidermis samples, to phosphate buffer saline (PBS) as negative control or to the positive control (5% sodium dodecyl sulfate, SDS; solution in distilled water).

Cell viability was determined with MTT (3-[4,5-dimethylthiazol-2-yl]-2,5-diphenyltetrazolium bromide) assay. Tissues were transferred to wells containing 2 mL of a 0.3 mg/mL MTT solution and incubated for 3 h (37°C, 5% CO_2_, 95% humidified atmosphere). After incubation, acidic isopropanol (0.5 mL/tube) was added to the epidermis tissues to extract the intracellular formazan.

The tubes were incubated for 4 h in dark with periodic vortexing and centrifuged, and a duplicate of 200 *μ*L was transferred to a 96-well flat-bottom microtitre plate. Absorbance was read at 570 nm with acidified isopropanol as blank and viability was calculated considering 100% for the negative control.

### 2.6. Human Repeat Insult Patch Test

A safety evaluation study was performed on SilEmulsion, using a Marzulli and Maibach [27] Human Repeated Insult Patch Test (HRIPT) protocol. In brief, the product was applied on the back of 50 healthy volunteers who gave informed written consent. Subjects with dermatological or other medical or physical conditions precluding topical application of the test material were excluded, along with pregnant and nursing women. Product was applied during 3 consecutive weeks as a set of 9 consecutive patches (Finn Chamber standard) always on the same area. The product was applied on day 1 and removed on day 3; an observation was performed and a new patch was then applied. This new patch was removed on day 5; a new observation was performed and a new patch was applied until day 8. The procedure continues until day 22. This ends the induction phase of the study.

At the product site, an occlusive patch containing 20 mg of the SilEmulsion was applied to the left side of the back where it remained for 48 hours. After that period, the patch was removed, the skin was evaluated, and a new patch was applied. Reactions after patching were scored according to International Contact Dermatitis Research Group (ICDRG) [28].

A 2-week rest period was followed without application of the test material. During the challenge period, new patches were prepared and fixed in the same manner as in the induction period but also on the right side of the back (i.e., a virgin site).

The patches were removed after 48 hours and scoring of skin reactions was performed in the same manner as before at 48, 72, and 96 hours after patching, using the same ICDRG scoring system.

The use of HRIPT studies for the evaluation of the SilEmulsion was submitted to the local Ethical Committee and respected the Helsinki Declaration to comply with good clinical practices. The study also complies with the Agence Francaise de Sécurité Sanitaire des Produits de Santé regulation on the performance of HRIPT studies on cosmetic products, which intends to guarantee that all the technical questions are evaluated meticulously during the application of the product in humans.

The study was conducted under the supervision of a dermatologist who participated in the evaluation of irritation/allergic reactions to the SilEmulsion.

### 2.7. Biological Effects

The transepidermal water loss (TEWL), epidermal capacitance, and skin surface lipids for the SilEmulsion were evaluated with a TEWAMETER TM 210, Corneometer CM 820 and a Sebumeter SM 810 (C + K Electronics GmbH, Germany), respectively, for a period of 21 days. A uniform volunteers panel was chosen (*n* = 10, young healthy females, 18–25 years old, the same professional activity), and subjects were included in the study after written and informed consent. The formulation was applied in the forearm and the results were compared with a defined control area (anatomically equivalent) on the same forearm with any treatment. Data were compared using a two-way ANOVA, comparing the SilEmulsion with the control area along the time (95% confidence level). Results are expressed as mean ± standard deviation (SD). 

Measurements were performed under standardized conditions, at room temperature.

### 2.8. Data Analysis

The data was analyzed using the ANOVA test (KaleidaGraph, version 4.0, Synergy Systems) and expressed as the mean ± SD; *P* < 0.05 was considered to be statistically significant.

## 3. Results and Discussion

### 3.1. Physical and Microbiological Stability of the SilEmulsion

The SilEmulsion was transparent and uniform in appearance. 

The pH (Table 2) did not significantly vary over time. The acidity of the skin ranges from pH 4 to 6; thus this result makes the formulation suitable for topical application [29]. Moreover, the pH value is in accordance with the maximum stability found for corticoids in aqueous solutions [30]. The SilEmulsion has suitable physical and chemical properties for the inclusion of a corticoid.

As demonstrated in Table 2, the viscosity of the SilEmulsion increased during the first months. This increase in viscosity was caused due to the swelling of the PEG chains in the solvent, decreasing the free water. The microbiological studies (Table 3) showed that the results were within the recommended limits of the specifications. These results indicate that the SilEmulsion is physically and microbiologically stable during at least 12 months.

### 3.2. Safety Assessment of the SilEmulsion

#### 3.2.1. Hazard Identification

It is important to know about the physical and chemical properties of each ingredient (Table 4) to predict the extend of permeation through the skin [31]. The chemical and physical properties and hazards of chemical compounds are precise and constant. In contrast, the properties of the same chemical in complex mixtures can vary considerably. The chemical structures of the ingredients used in the SilEmulsion, namely, the surfactant and coemulsifier are very complex [3]; thus, it is difficult to predict interactions between them. Nevertheless, it is accepted that the safety of a cosmetic product is determined based on the safety assessment of its ingredients [7].

Molecules must be in the liquid form to get absorbed through the skin; molecules in the solid state are not absorbed.

As a general rule, chemicals with a molecular weight greater than 500 Da do not penetrate the skin. This is known as the “rule of 500” [32]. This upper limit on molecular size mainly results from the physical arrangement of lipids between adjacent corneocytes of the SC. Considering the molecular weight of the ingredients presented in Table 4, it can be concluded that both polymers (HPMC and PVM/MA) and the silicone-based surfactant will not be able to penetrate the SC.

The relationship between solubility and the rate of skin absorption stems primarily from the ability of a chemical to partition into the SC. If a chemical is excessively hydrophilic, it will not partition into the predominantly lipid environment of the SC. In contrast, if a chemical is too strongly lipophilic, it will readily partition into the SC but will not partition out into the predominantly hydrophilic environment of the underlying epidermal tissue. Thus, in order to penetrate the skin, the solubility of a chemical requires a balance between these two extremes. In general, a partition coefficient (Log *P*) between 1 and 3 is considered to be optimal for skin absorption [33]. Considering the molecular weight and the Log *P* values, the ingredients which are most likely to penetrate into the SC are cetrimide and 1,5-pentanediol.

The biological safety evaluation requires that cytotoxicity, sensitization, and irritation or intracutaneous reactivity are determined and the risk of chronic toxicity, carcinogenicity, reproductive/development toxicity, or other organ-specific toxicities based on specific nature and duration of exposure of the product is assessed (Table 5) [34].

Emulsifiers are of particular concern due to their potential to cause irritation [35, 36] and because they have the potential to act as penetration enhancers by decreasing surface tension and conditioning the SC and hence may enable or enhance diffusion of other molecules through the skin [37]. The main emulsifier present in the SilEmulsion is a silicone-based emulsifier containing polyethylene glycol (PEG) chains as the hydrophilic part and medium-chain triglycerides as the lipophilic part. Due to the absence of data in the literature for this emulsifier, we decompose this ingredient into three parts: PEG, dimethicone, and medium-chain triglycerides, and we assessed the safety profile of the individual ingredients.

PEGs and PEG fatty esters were not or very slightly irritating to the skin of rabbits and humans [38]. However, independent of the erythema, increased TEWL was induced by some of the emulsifiers, indicating an invisible impairment of the SC barrier function [8]. Clinical and animal absorption studies reported that dimethicone was not absorbed following oral or dermal exposure. Dimethicone was not acutely toxic following oral exposure. No adverse reactions were found in rabbits following short-term dermal dosing with 6% to 79% dimethicone. Most dermal irritation studies using rabbits classified dimethicone as a minimal irritant. Dimethicone (tested undiluted and at 79%) was not a sensitizer in four assays using mice and guinea pigs. Moreover, it was not a sensitizer at 5.0% in a clinical repeated insult patch test using 83 panelists. Most ocular irritation studies using rabbits classified dimethicone as a mild-to-minimal irritant. Dimethicone was tested in numerous oral-dose (using rats) and dermal-dose (using rats, rabbits, and monkeys) reproductive and developmental toxicity studies. Dimethicone was negative in all genotoxicity assays. It was negative in both oral (tested at 91%) and dermal (tested at an unknown concentration) dose carcinogenicity assay using mice [39].

Medium-chain triglycerides exhibit very low levels of toxicity in a variety of laboratory animals and in humans when administered orally, parenterally, or by the dermal route [40].

Based on these results concerning PEGs and dimethicone and medium-chain triglycerides, we can predict that the bis-PEG/PPG-16/16 PEG/PPG-16/16 dimethicone (and) caprylic/capric triglyceride pose no consumer risk in the concentration used.

Concerning the co-emulsifier, it was demonstrated that glyceryl monoesters have little acute or short-term toxicity in animals, and no toxicity was noted following chronic administration of a mixture consisting mostly of glyceryl di- and monoesters. Glyceryl laurate was not classified as ocular irritant in rabbits. Undiluted glyceryl monoesters may produce minor skin irritation, especially in abraded skin, but, in general, these ingredients are not irritating at concentrations used in cosmetics. Glyceryl monoesters are neither sensitizers nor photosensitizers. At concentrations higher than those used in cosmetics, glyceryl laurate did cause moderate erythema in HRIPT studies. Based on these data, the Cosmetic Ingredient Review Expert Panel found that these glyceryl monoesters are safe as cosmetic ingredients in the present practices of use and concentration [37].

Based on these data the ingredients of special concerns are cetrimide and 1,5-pentanediol because they present suitable physical characteristics to penetrate the skin; the glycol is present in the formulation in a relatively high concentration and cetrimide has been shown to be irritant to the skin and a sensitizer.

#### 3.2.2. Exposure Assessment

SilEmulsion is intended for use on intact skin of adults. It can be used as an adjuvant in corticoid therapy. It is applied to the affected area in the desired quantity once or twice a day with a soft massage to enhance the product absorption.

It will be supplied for use as a leave-on cosmetic product which is intended to stay in prolonged contact with the skin.

According to the Scientific Committee on Consumer Safety [25], the human surface area is 15670 cm^2^. The SilEmulsion will be considered as a body cream; thus, according the Scientific Committee on Consumer Safety, the estimated daily amount applied for a body cream is 7.82 g/day and the frequency of application is 2.28 times per day which is translated in a daily exposure of 123.2 bw/day (Table 6).

From Table 6, it can be seen that the estimated SED from the ingredients present in the SilEmulsion ranged from 0.09 to 12.32 mg/kg bw/day for cetrimide and 1,5-pentanediol, respectively. The SED is a tool to predict the systemic availability of a cosmetic substance; however, this relationship is not straightforward. In the absence of dermal absorption studies, the worst-case scenario of 100% of dermal absorption should be taken into consideration [25]. Thus, the estimated SED is overestimated, which means that, for example, for cetrimide which is a good candidate for dermal absorption, (Table 4) the SED may be indeed 0.09 mg/kg bw/day but, for HPMC, it is expected to be much lower. The SED values should be taken as orientative values and must be analyzed regarding the overall chemical, physical, and hazard data.

#### 3.2.3. Dose-Response Assessment

The NOAEL is mainly derived from repeated-dose animal studies (90 day, developmental toxicity studies, etc.).

As far as the determination of critical effects in repeated-dose toxicity studies is concerned, the available repeated-dose toxicity data should be evaluated in detail for a characterization of the health hazards upon repeated exposure. The NOAEL values found out for cetrimide and 1,5-pentanediol were 20 and 450 mg/kg bw/day, respectively [14, 41].

#### 3.2.4. Risk Characterisation

The MoS is used to extrapolate from a group of test animals to an average human being and subsequently from average humans to sensitive subpopulations. The WHO proposes a minimum value of 100, and it is generally accepted that the MoS should at least be 100 to declare a substance safe for use [25].

The value of 100 consists of a factor 10 for the extrapolation from animal to man and another factor 10 taking into account the interindividual variations within the human population.

However, in the majority of MoS calculations, this dermal exposure figure is compared to an oral NOAEL value, which corresponds to the amount that has been administered orally, though not necessarily to the actual systemic availability of the compound after oral administration.

The MoS for the two ingredients of special concerns (cetrimide and 1,5-pentanediol) were calculated according to (2). The MoS value obtained for cetrimide was 222.22 which is above the threshold value of 100 suggesting that the ingredient under study can be considered to pose no consumer risks or systemic toxicity effects. Concerning 1,5-pentanediol the value obtained was 36.53; however, it should be emphasized that this is a very conservative approach. In fact, the actual safety margins of cosmetic ingredients tend to be higher than theoretical values, since calculated MoS data represents a worst-case scenario. For example, a skin penetration of 100% was assumed which may not correspond to the penetration in reality. In this case *in vitro* and *in vivo* tests will be useful to decide about the safety of this ingredient.

### 3.3. EpiSkin Assay

The safe topical use of the SilEmulsion was tested on reconstituted human epidermis. The EpiSkin model mimics morphologically and biochemically the living skin and is useful to classify skin irritants which can cause decrease in cell viability, evaluated by an MTT assay [42]. The tissue viability, measured as optical density at 570 nm by the MTT assay and calculated as percentage of cytotoxicity compared to the negative control (PBS), was 92 ± 6.0%, whereas in the positive control (SDS) it was 30.0 ± 4%. A product is considered an irritant when viability is reduced by 50%.

The absence of skin-irritant effects at the concentrations tested indicated that SilEmulsion could be safe for topical use.

### 3.4. Human Repeat Insult Patch Tests

The experimental conditions adopted in this study allowed the creation of occlusive conditions. According to the Marzulli and Maibach protocol [27], the products intended to be used as leave-on products should have an increased exposure only obtained with an occlusive patch. The occlusion favors the permeation through the skin, which allows an easy viewing of irritative reactions.

During the HRIPT study, no reactions were observed in the initial 3-week contact or after the final challenge contact.

Therefore, the repeated application of the product did not induce any sensitization on the skin of the volunteers and the SilEmulsion presented very good skin compatibility.

### 3.5. Biological Effects

**Figure 1:**
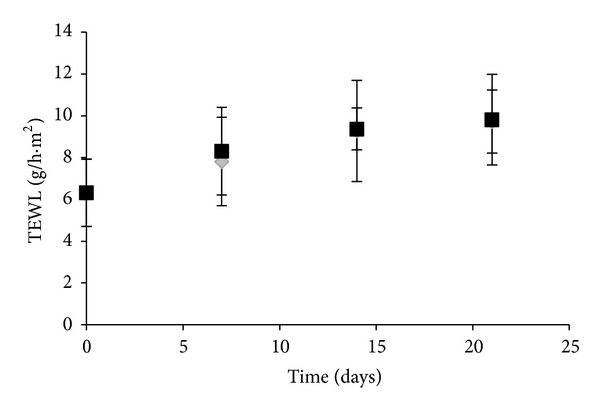
Comparison of TEWL during 21 days between SilEmulsion (black bars) and control (grey bars) (mean ± SD, *n* = 10).

The skin is often exposed to surface-active agents like soaps, which may affect the skin barrier. Differences in the effects of surfactants have been investigated previously, for example, using biophysical instruments [8, 43]. These investigations show that surfactants exert strong effects in experimental settings. SLS, a surfactant with a carbon chain length of 12, is ranked as the most irritating [44]. An increased TEWL is a sensitive measure of barrier damage [44, 45] and an indication of the skin permeability [46].  Figure 1 shows the comparison between SilEmulsion and control area in terms of TEWL during 21 days. The SilEmulsion did not significantly increased TEWL compared to the control.

SC water retention and skin surface lipids properties are crucial factors in keeping the skin supple and flexible and influence skin permeability to molecules. The methodological procedure chosen allowed the identification of positive results regarding skin water dynamics, expressed in terms of corneometry changes and skin lipids expressed in terms of sebum (Table 7).

The *in vivo* studies for human skin hydration showed a slight increase after application of SilEmulsion when compared to the control area (*P* > 0.05). The principal mechanisms of hydration are humectancy, emolliency, and occlusion. The hydration provided by SilEmulsion is mainly attributed to humectants (1,5-pentanediol) and emollients (PEG-based surfactants, isopropyl myristate, and C12–15 alkyl benzoate). In fact, humectants promote water retention within the SC, whereas emollients smooth the skin by filling the spaces between skin flakes and adding semiocclusive activity which contributes to SC hydration [47]. Occlusive agents increase moisture levels by providing a physical barrier to epidermal water loss; petrolatum, waxes, and silicones are occlusive substances. The silicone-based surfactant present in the SilEmulsion was not sufficient to confer occlusive properties to the emulsion, since the TEWL values were not different from the control area.

On the other hand, a drastic increase in the skin lipids occurred after application of SilEmulsion (Table 7). It was demonstrated that lipids of mineral or plant origin may partially substitute for skin lipids and improve both the feel and function of a lipid-depleted skin [48]. Although barrier function requires cholesterol, free fatty acids, and ceramides, applications of exogenous nonphysiologic lipids seems to contribute to the barrier function. It was demonstrated that petrolatum remains restricted to the SC and produced more rapid improvement in barrier function than the physiologic lipids. These observations are due to that the physiologic lipids only improve barrier recovery after transport to subjacent nucleated layers, followed by internalization, apparent transport to the distal Golgi apparatus, and incorporation into nascent lamellar bodies [49].

## 4. Conclusion

Considering the composition of the product and the physicochemical characteristics of the ingredients, the physical and microbiological quality and stability of the SilEmulsion, the toxicological profile of the ingredients, the risk characterization, and the *in vitro* and *in vivo* results, the SilEmulsion can be considered safe in the normal and reasonably foreseeable use. Additionally, SilEmulsion is demonstrated to contribute to restore the skin barrier by increasing the amount of lipids within the skin. A suitable equilibrium between safety and biological effects was demonstrated.

## Figures and Tables

**Table 1:** Qualitative and quantitative composition of the SilEmulsion.

INCI name	Main functions/additional functions	Concentration (%)
Bis-PEG/PPG-16/16 PEG/PPG-16/16 dimethicone (and) caprylic/capric triglyceride	Nonionic surfactant/sensorial modifier	5.0
PEG-20 glyceryl laurate	Nonionic coemulsifier	4.0
Isopropyl myristate	Oil internal phase/penetration enhancer	5.0
C12-15 alkyl benzoate	Oil internal phase	5.0
HPMC	Thickening agent/polymeric emulsifier	2.0
PVM/MA	Thickening agent/polymeric emulsifiers	0.3
Cetrimide BP	Preservative/cationic surfactant	0.075
1,5-Pentanediol	Solubilizer	10.0
Water	Aqueous external phase	68.625

**Table 2:** Stability test results for SilEmulsion during 12 months at 25°C, 30°C, and 40°C.

Conditions of storage	25°C		30°C		40°C	
Time (months)	pH	Apparent viscosity (Pa·s)	pH	Apparent viscosity (Pa·s)	pH	Apparent viscosity (Pa·s)
0	4.38	12417	4.38	12417	4.38	12417
1	4.38	17486	4.49	19886	4.16	16437
3	4.45	20396	4.48	18506	4.20	16796
6	4.47	20606	4.31	16546	4.34	14817
12	4.44	22200	4.20	19620	4.43	17280

**Table 3:** Microbiological stability of the SilEmulsion.

Time (0, 1, 3, 6, 12 months)	Total aerobic microbial count		Yeast/mould count	*E. coli* *P. aeruginosa*
	30°C	37°C		*S. aureus *
25°C	Conform	Conform	Conform	Conform
30°C/75% RH	Conform	Conform	Conform	Conform
40°C/75% RH	Conform	Conform	Conform	Conform

**Table 4:** Physical and chemical properties of the ingredients presented in the SilEmulsion.

INCI name	CAS number	Molecular weight (g/mol)	Impurities	Log *P*_ow_*
Aqua	7732-18-5	18.02	n.a.	—
Bis-PEG/PPG-16/16 PEG/PPG-16/16 dimethicone (and) caprylic/capric triglyceride	n.a.	>10000 [10]	n.a.	n.a.
PEG-20 glyceryl laurate	*59070-56-3 *	362.50 [11]	Ethyleneoxide <1 ppm Dioxane <5 ppm	3.70 [11]
Isopropyl myristate	110-27-0	270.45 [11]	Ash <0.10% Water content <0.10%	7.02 [11]
C12-15 alkyl benzoate	68411-27-8	290.44 [11]		7.16 [11]
Hidroxypropyl methylcellulose	9004-65-3	>13000 <200000 [12]		−2.34 [11]
PVM/MA decadiene crosspolymer	136392-67-1	>1000000 [13]	Cyclohexane and ethyl acetate <0.75% Maleic anhydride negative	n.a.
Cetrimide	1119-97-7	364.45 [11]	Free amines <0.15% Amine HBr <0.3% Sulphated ash <0.5%	1.86 [11]
1,5-Pentanediol	111-29-5	104.15		0.58 [14]

*partition coefficient between n-octanol and water.

**Table 5:** Summary of the biological safety of the ingredients.

INCI name	Acute toxicity	Dermal irritation	Ocular irritation	Sensitization	Genotoxicity/ carcinogenicity	References
Bis-PEG/PPG-16/16 PEG/PPG-16/16 dimethicone (and) caprylic/capric triglyceride	n.a	n.a	n.a	n.a	n.a	—

PEG-20 glyceryl laurate	Rat (oral) LD_50_ > 48 mL/kg	Non irritant	Rabbit: non irritant	n.a	n.a	[15]

Isopropyl myristate	Rat (oral) LD_50_ > 5000 mg/kg	Rabbit (undiluted): mild irritant	Rabbit: minimally irritant	Guinea pig: nonsensitizer Human: nonsensitizer	n.a	[16, 17]

C12-15 alkyl benzoate	Rat (oral) LD_50_ > 2000 mg/kg Rabbit (dermal) LD_50_ > 2000 mg/kg	Rabbit: non irritant	Rabbit: non irritant	Guinea pig: non sensitizer	n.a	[18, 19]

Hidroxy propyl methyl cellulose	Oral LD_50_ > 10000 mg/kg	Can cause irritation	Can cause irritation	Guinea pig: non sensitizer	n.a	[12]

PVM/MA decadiene crosspolymer	Rat (oral) LD_50_ > 1500 mg/kg Rat (oral), 1% in solution LD_50_ > 5000 mg/kg	Rabbit: slightly irritant	May cause irritation	Human patch test: non sensitizer (2% gel)	*In vitro* gene mutation in bacteria: negative	[20]

Cetrimide	Rat (oral) LD_50_ > 400 < 600 mg/kg	Rabbit: irritant	Potent irritant	Sensitizer	*Salmonella Typhimurium*: negative	[21]

1,5-Pentanediol	Rat (oral) LD_50_ 10000 mg/kg Rabbit (dermal) LD_50_ > 19800 mg/kg	Rabbit: non irritant	Rabbit: non irritant	n.a	Ames test: negative	[22]

**Table 6:** Exposure data of formulation ingredients.

Ingredient	Daily exposure (mg/kg bw/day)	% in the final product	Dermal absorption*	SED (mg/kg bw/day)
Bis-PEG/PPG-16/16 PEG/PPG-16/16 dimethicone (and) caprylic/capric triglyceride	123.2	5.0	100	6.16
PEG-20 glyceryl laurate	123.2	4.0	100	4.93
Isopropyl myristate	123.2	5.0	100	6.16
C12-15 alkyl benzoate	123.2	5.0	100	6.16
HPMC	123.2	2.0	100	2.46
PVM/MA	123.2	0.3	100	0.37
Cetrimide	123.2	0.075	100	0.09
1,5-Pentanediol	123.2	10.0	100	12.32

*When no permeation data is available, the value considered is 100%.

**Table 7:** Comparison of skin hydration values in terms of capacitance and skin surface lipids during 21 days between SilEmulsion and control area (mean ± SD, *n* = 10).

	0	7	14	21	*P* value
Corneometry (AU)					
SilEmulsion	41.17 ± 5.16	48.08 ± 4.68	49.58 ± 3.92	48.92 ± 5.38	0.064
Control	41.08 ± 4.50	43.83 ± 3.71	44.42 ± 3.94	43.83 ± 4.95	
Skin lipids (*µ*g/cm^2^)					
SilEmulsion	0.67 ± 0.89	34.08 ± 7.94	32.50 ± 6.93	28.92 ± 7.11	<0.001
Control	0.67 ± 0.89	0.42 ± 0.51	0.58 ± 0.90	0.75 ± 0.75	

## Abbreviations

BW: Body weight
DA: Dermal absorption
HPMC: Hydroxypropyl methylcellulose
HRIPT: Human repeated insult patch test
ICDRG: International contact dermatitis research group
MoS: Margin of safety
MTT: 3-[4,5-Dimethylthiazol-2-yl]-2,5-diphenyltetrazolium bromide
NOAEL: No observed adverse effect level
PBS: Phosphate buffer saline
PEG: Polyethylene glycol
PVM/MA: Methyl vinyl ether/maleic anhydride copolymer cross-linked with decadiene
SC: *Stratum corneum*
SD: Standard deviation
SDS: Sodium dodecyl sulfate
SED: Systemic exposure dose
TEWL: Transepidermal water loss
TG: Topical glucocorticoids.

## References

[1] Raposo S, Simões S, Almeida AJ, Ribeiro HM (2013). Advanced systems for glucocorticoids' dermal delivery. *Expert Opinion on Drug Delivery*.

[2] Eccleston GM (1997). Functions of mixed emulsifiers and emulsifying waxes in dermatological lotions and creams. *Colloids and Surfaces A*.

[3] Raposo S, Salgado A, Eccleston G, Urbano M, Ribeiro HM (2013). Cold processed oil-in-water emulsions for dermatological purpose: formulation design and structure analysis. *Pharmaceutical Development and Technology*.

[4] EU Council Directive 76/768/EEC of 27 July 1976 on the approximation of the laws of the Member States relating to cosmetic products.

[5] European Parliament and Council Regulation (EC) No. 1223/2009 of the European. Parliament and of the Council of 30 November 2009 on cosmetic products (recast)—text of importance for the EEA (Official Journal of the European Union L 342/59, 22 December 2009). http://eur-lex.europa.eu/LexUriServ/LexUriServ.do?uri=OJ:L:2009:342:0059:0209:EN:PDF.

[6] Rothe H, Fautz R, Gerber E (2011). Special aspects of cosmetic spray safety evaluations: principles on inhalation risk assessment. *Toxicology Letters*.

[7] Pauwels M, Rogiers V (2010). Human health safety evaluation of cosmetics in the EU: a legally imposed challenge to science. *Toxicology and Applied Pharmacology*.

[8] Bárány E, Lindberg M, Lodén M (2000). Unexpected skin barrier influence from nonionic emulsifiers. *International Journal of Pharmaceutics*.

[9] Nohynek GJ, Antignac E, Re T, Toutain H (2010). Safety assessment of personal care products/cosmetics and their ingredients. *Toxicology and Applied Pharmacology*.

[10] Evonik, 2008 Emulsifiers for skin care application. http://www.finecon.sk/userfiles/file/broschuere_pc_3.pdf.

[11] VCCLAB, Virtual Computational Chemistry Laboratory. http://www.vcclab.org/.

[12] Burdock GA (2007). Safety assessment of hydroxypropyl methylcellulose as a food ingredient. *Food and Chemical Toxicology*.

[13] National industrial chemicals notification and assessment scheme Full public report on poly (maleic anhydridemethyl vinyl ether) by 1,9-decadiene. http://www.docin.com/p-393512288.html.

[14] Wagner P Inert Reassessment—2-methyl-2,4-pentanediol.

[15] Tagat L2, MSDS http://corporate.evonik.com/en/Pages/default.aspx.

[16] Tegosoft M, MSDS http://corporate.evonik.com/en/Pages/default.aspx.

[17] Cosmetic ingredient review (CIR) (1982). Final Report on the Safety Assessment of Myristyl Myristate and Isopropyl Myristate. *International Journal of Toxicology*.

[18] Tegosoft TN MSDS http://msds.consumer-specialties.evonik.com/msds/site6/e/result/report.jsp.

[19] Cosmetic ingredient review (CIR) (2010). Expert Panel Meeting. *Green Book*.

[20] Stabileze QM, MSDS http://www.ashland.com/.

[21] SCCS, Scientific Committee on Consumer Safety Opinion on alkyl (C_16_, C_18_, C_22_) trimethylammonium chloride.

[22] 1,5-pentanediol MSDS http://www.sigmaaldrich.com/MSDS/MSDS/DisplayMSDSPage.do?country=PT&language=pt&productNumber=68336&brand=FLUKA&PageToGoToURL=http%3A%2F%2Fhttp://www.sigmaaldrich.com%2Fcatalog%2Fproduct%2Ffluka%2F68336%3Flang%3Dpt.

[23] CPMP/QWP/122/02, rev 1 Guideline on stability testing: stability testing of existing active substances and related finished products.

[24] Farmacopeia Portuguesa

[25] SCCS, European Commission Health and Consumer Protection, Directorate-General for Health and Consumers. Scientific Committee on Consumer Safety “The SCCS's Notes of Guidance For Testing of Cosmetic Ingredients and their Safety Evaluation”. http://ec.europa.eu/health/scientific_committees/consumer_safety/docs/sccs_s_004.pdf.

[27] Marzulli FN, Maibach HI (1976). Contact allergy: predictive testing in man. *Contact Dermatitis*.

[28] Fregert S, Bandmann H (1975). *International Contact Dermatitis Research Group Patch Testing*.

[29] Lodén M (2003). Role of topical emollients and moisturizers in the treatment of dry skin barrier disorders. *American Journal of Clinical Dermatology*.

[30] Teng XW, Cutler DC, Davies NM (2003). Degradation kinetics of mometasone furoate in aqueous systems. *International Journal of Pharmaceutics*.

[31] Potts RO, Guy RH (1992). Predicting skin permeability. *Pharmaceutical Research*.

[32] Bos JD, Meinardi MMHM (2000). The 500 Dalton rule for the skin penetration of chemical compounds and drugs. *Experimental Dermatology*.

[33] Brain KR, Chilcott RP, Chilcott RP, Price S (2008). Physicochemical factors affecting skin absorption. *Principles and Practice of Skin Toxicology*.

[34] Pauwels M, Rogiers V (2007). Database search for safety information on cosmetic ingredients. *Regulatory Toxicology and Pharmacology*.

[35] Djekic L, Primorac M (2008). The influence of cosurfactants and oils on the formation of pharmaceutical microemulsions based on PEG-8 caprylic/capric glycerides. *International Journal of Pharmaceutics*.

[36] Zanatta CF, Ugartondo V, Mitjans M, Rocha-Filho PA, Vinardell MP (2008). Low cytotoxicity of creams and lotions formulated with Buriti oil (*Mauritia flexuosa*) assessed by the neutral red release test. *Food and Chemical Toxicology*.

[37] (CIR) Cosmetic ingredient review (2004). Final report of the amended safety assessment of Glyceryl Laurate, Glyceryl Laurate SE, Glyceryl Laurate/Oleate, Glyceryl Adipate, Glyceryl Alginate, Glyceryl Arachidate, Glyceryl Arachidonate, Glyceryl Behenate, Glyceryl Caprate, Glyceryl Caprylate, Glyceryl Caprylate/Caprate, Glyceryl Citrate/Lactate/Linoleate/Oleate, Glyceryl Cocoate, Glyceryl Collagenate, Glyceryl Erucate, Glyceryl Hydrogenated Rosinate, Glyceryl Hydrogenated Soyate, Glyceryl Hydroxystearate, Glyceryl Isopalmitate, Glyceryl Isostearate, Glyceryl Isostearate/Myristate, Glyceryl Isostearates, Glyceryl Lanolate, Glyceryl Linoleate, Glyceryl Linolenate, Glyceryl Montanate, Glyceryl Myristate, Glyceryl Isotridecanoate/Stearate/Adipate, Glyceryl Oleate SE, Glyceryl Oleate/Elaidate, Glyceryl Palmitate, Glyceryl Palmitate/Stearate, Glyceryl Palmitoleate, Glyceryl Pentadecanoate, Glyceryl Polyacrylate, Glyceryl Rosinate, Glyceryl Sesquioleate, Glyceryl/Sorbitol Oleate/Hydroxystearate, Glyceryl Stearate/Acetate, Glyceryl Stearate/Maleate, Glyceryl Tallowate, Glyceryl Thiopropionate, and Glyceryl Undecylenate. *International Journal of Toxicology*.

[38] Fruijtier-Pölloth C (2005). Safety assessment on polyethylene glycols (PEGs) and their derivatives as used in cosmetic products. *Toxicology*.

[39] Andersen FA (2003). Final report on the safety assessment of stearoxy dimethicone, dimethicone, methicone, amino bispropyl dimethicone, aminopropyl dimethicone, amodimethicone, amodimethicone hydroxystearate, behenoxy dimethicone, C24-28 alkyl methicone, C30-45 alkyl methicone, C30-45 alkyl dimethicone, cetearyl methicone, cetyl dimethicone, dimethoxysilyl ethylenediaminopropyl dimethicone, hexyl methicone, hydroxypropyldimethicone, stearamidopropyl dimethicone, stearyl dimethicone, stearyl methicone, and vinyldimethicone. *International Journal of Toxicology*.

[40] Traul KA, Driedger A, Ingle DL, Nakhasi D (2000). Review of the toxicologic properties of medium-chain triglycerides. *Food and Chemical Toxicology*.

[41] Blackburn K, Stickney JA, Carlson-Lynch HL, McGinnis PM, Chappell L, Felter SP (2005). Application of the threshold of toxicological concern approach to ingredients in personal and household care products. *Regulatory Toxicology and Pharmacology*.

[42] Diaz-Reinosoa B, Mourea A, Dominguez H, Parajo JC (2011). Membrane concentration of antioxidants from Castanea sativa leaves aqueous Extracts. *Chemical Engineering Journal*.

[43] Bárány E, Lindberg M, Lodén M (1999). Biophysical characterization of skin damage and recovery after exposure to different surfactants. *Contact Dermatitis*.

[44] Wilhelm K-P, Freitag G, Wolff HH (1994). Surfactant-induced skin irritation and skin repair: evaluation of the acute human irritation model by noninvasive techniques. *Journal of the American Academy of Dermatology*.

[45] Van Der Valk PGM, Crijns MC, Nater JP, Bleumink E (1984). Skin irritancy of commercially available soap and detergent bars as measured by water vapour loss. *Dermatosen in Beruf und Umwelt*.

[46] Lévêque JL, Lévêque JL (1989). Measurement of transepidermal water loss. *Cutaneous Investigation in Health and Disease. Noninvasive Methods and Instrumentation*.

[47] Kraft JN, Lynde CW (2005). Moisturizers: what they are and a practical approach to product selection. *Skin Therapy Letter*.

[48] Rudolph R, Kownatzki E (2004). Corneometric, sebumetric and TEWL measurements following the cleaning of atopic skin with a urea emulsion versus a detergent cleanser. *Contact Dermatitis*.

[49] Mao-Qiang M, Brown BE, Wu-Pong S, Feingold KR, Elias PM (1995). Exogenous nonphysiologic vs physiologic lipids: divergent mechanisms for correction of permeability barrier dysfunction. *Archives of Dermatology*.

